# A qualitative study exploring women’s beliefs about physical activity after stillbirth

**DOI:** 10.1186/1471-2393-14-26

**Published:** 2014-01-17

**Authors:** Jennifer L Huberty, Jason Coleman, Katherine Rolfsmeyer, Serena Wu

**Affiliations:** 1 Exercise and Wellness, Arizona State University, Phoenix, AZ 85004, USA; 2 Health, Physical Education, and Recreation, University of Nebraska Omaha, Omaha, NE 68182, USA; 3 Department of Obstetrics and Gynecology, New York Institute of Technology, Good Samaritan Hospital Medical Center, West Islip, NY 11795, USA; 4 Department of Public Health, Los Angeles County, CA 90012, USA

**Keywords:** Stillbirth, Loss, Physical activity, Women, Health

## Abstract

**Background:**

Research provides strong evidence for improvements in depressive symptoms as a result of physical activity participation in many populations including pregnant and post-partum women. Little is known about how women who have experienced stillbirth (defined as fetal death at 20 or more weeks of gestation) feel about physical activity or use physical activity following this experience. The purpose of this study was to qualitatively explore women’s beliefs about physical activity following a stillbirth.

**Methods:**

This was an exploratory qualitative research study. Participants were English-speaking women between the ages of 19 and 44 years who experienced a stillbirth in the past year from their recruitment date. Interviews were conducted over the phone or in-person based on participants’ preferences and location of residence and approximately 30–45 minutes in length.

**Results:**

Twenty-four women participated in the study (*M* age = 33 ± 3.68 years; *M* time since stillbirth = 6.33 ± 3.06 months). Women’s beliefs about physical activity after stillbirth were coded into the following major themes: barriers to physical activity (emotional symptoms and lack of motivation, tired, lack of time, guilt, letting go of a pregnant body, and seeing other babies), benefits to physical activity (feeling better emotionally/mentally, helping women to cope or be therapeutic), importance of physical activity (working through grief, time for self), motivators for physical activity (body shape/weight, health, more children, be a role model, already an exerciser). Health care providers and their role in physical activity participation was also a major theme.

**Conclusions:**

This is the first study to qualitatively explore beliefs about physical activity in women after a stillbirth. Women who have experienced stillbirth have unique beliefs about physical activity related to their experience with stillbirth. Findings from this study may help to improve the health and quality of life for women who have experienced stillbirth by utilizing physical activity as a strategy for improving depressive symptoms associated with experiencing a stillbirth. Future research in this area is highly warranted.

## Background

Stillbirth has been established as a psychological trauma and is one of the most common adverse pregnancy outcomes in the United States [1]. Defined as fetal death at 20 or more weeks of gestation, stillbirth occurs 100 times per day and in one in every 150 pregnancies, totaling approximately 25,894 per year [2,3]. The labor, birth, and postpartum periods of women who experience stillbirth are physically similar to those of women who give birth to live, healthy babies; however, the negative effects are significantly greater [4]. Consequently, stillbirth presents a threefold increased risk of postpartum depression when compared with live, healthy births [5,6], and symptoms (e.g., perceived stress, anxiety, and sleep quality) may last for years or decades [1,4,6]. Depression may contribute to health problems, including weight retention or gain, increased risk of chronic disease (e.g., heart disease, diabetes), premature mortality, and poor quality of life [7,8]. These symptoms may continue with subsequent pregnancies and after the birth of a healthy child [6].

Research provides strong evidence for improvements in depressive symptoms as a result of physical activity participation in many populations, including pregnant and post-partum women [9,10]. Women who are physically active during their pregnancy experience fewer depressive symptoms as compared to physically inactive pregnant women [11-13]. Studies also suggest that after giving birth, participation in leisure-time physical activity, regardless of activity level during pregnancy, may improve depressive symptoms and positively effect well-being [12,13]. Because women who experience stillbirth undergo pregnancy-related physiological/psychological effects, in addition to suffering trauma-related negative psychological and emotional effects, physical activity may be a particularly important strategy to improve depressive symptoms in this population.

Little is known about how women who have experienced stillbirth feel about physical activity or use physical activity following this experience. Recently, there has been an increase in research to determine the perceptions and beliefs about physical activity in pregnant and post-partum women [14,15], but no studies exist in women who have experienced stillbirth. Post-partum women of live, healthy births report fatigue, lack of time, lack of family support, and lack of childcare as major barriers to physical activity [14]. Reasons women engage in physical activity post-partum include improvements in health and fitness (e.g., loss of baby weight, feeling fit) [14,15]. Because having a stillbirth has more negative effects as compared to having a live, healthy birth, the perceptions of physical activity during and after pregnancy in women who have had a stillbirth may be different.

Considering the elevated risk of depressive symptoms as compared to women of healthy live births, the potential of physical activity as a mechanism to improve these symptoms, and the implications physical activity may have on the mother’s health overall (especially in relation to subsequent children), research about how women who have experienced stillbirth perceive physical activity is needed. Therefore, the purpose of this study was to qualitatively explore the beliefs about physical activity in women after a stillbirth.

## Methods

This exploratory qualitative research study was conducted between August 2012 and May 2013. Human subjects approval was obtained from an Institutional Review Board and informed consent was attained from each participant prior to her participation. Women were eligible to participate if they were between the ages of 19 and 44 years, were English-speaking, and had experienced a stillbirth in the past year from the recruitment date. Women were recruited nationally by: 1) contacting women who had provided their contact information on a survey for a prior study, 2) distributing fliers to obstetrics and gynecology clinics and national stillbirth research and education foundations; 3) utilizing email listservs, electronic newsletters, websites, and Facebook; and 4) word of mouth.

A trained research assistant conducted interviews using a semi-structured interview guide by phone or in-person based on participants’ preferences and location of residence. Interviews were approximately 30–45 minutes in length and were recorded. The Health Belief Model [16], which predicts health behaviors by focusing on the attitudes and beliefs of individuals, guided the development of interview questions. For example, one of the questions asked was, “What emotions have you experienced since the loss of (child’s name)? Have these emotions altered your physical activity levels? If so, in what ways?” This question was developed to determine barriers to physical activity related to loss. Probes were added to explore new insights from previous interviews. Interview question topics and examples of questions for each topic are provided in Table 1.

**Table 1:** Examples of questions/definition for each topic

**Interview question topics**	**Example definition/questions**
Definition of physical activity	Any body movement that requires energy. Examples are walking, biking, gardening, and cleaning house.
Participation in physical activity	Are you currently participating in regular moderate intensity physical activity (equal to a brisk walk) for at least 30 minutes on most if not all days of the week?
Benefits of/Barriers to physical activity participation	What barriers have you encountered to participating in physical activity after losing (child’s name)?
	What emotions have you experienced since the loss of (child’s name)? Have these emotions altered your physical activity levels? If so, in what ways?
	Do you believe physical activity plays a role in mood after pregnancy? If yes, how?
Physical activity motivation	Tell me, if any, about your personal motivations to be physically active since you became pregnant with (child’s name).
	Have you ever thought about using physical activity to help you cope with your symptoms? If so, how effective would you say physical activity has been in helping you cope?
Importance of physical activity	Currently, how important is physical activity to you?
	How important is physical activity to you as it relates to your feelings of grief and loss?
Beliefs about the relationship between physical activity and health/weight	Recognizing that the loss of your much loved baby is often in the forefront of our mind, do you believe physical activity plays a role in weight loss after pregnancy? If so, how?
	Is physical activity important to you as it relates to weight loss?
Support for physical activity from health care provider	What kind of support/resources do you think you need to be physically active?
	What instructions were you given from your health care provider about physical activity after the loss of your baby?

### Analytical methods

Interviews were transcribed verbatim and analysis was conducted using NVivo 10 (QSR International, 2012). The lead researcher read each transcript and developed initial codes using an inductive process [17]. After initial coding, codes were added and refined based on a second reading of the transcripts and a codebook was created. Based on the codebook, a second researcher independently coded the data. Discrepancies in coding were discussed between the coders until consensus was achieved.

## Results

### Sample description

Forty-one women volunteered for the study and 40 women were contacted by the research team and asked to participate. Of the 81 women, 25 were ineligible (had been more than a year since their loss (n = 23) or their baby passed away 5+ weeks after being born (n = 2)), 29 were unreachable by phone or email, and 2 did not want to participate. Twenty-five women interviewed for the study and then one was excluded due to a rare chronic illness. A total of 24 women were included in data analysis for this study (*M* age = 33 ± 3.68 years; *M* time since stillbirth = 6.33 ± 3.06 months) with 50% reporting they were currently active. See Table 2 for participant demographics.

**Table 2:** Sociodemographics of participants

**Sociodemographics**	***n *** **(%)**
**Race/Ethnicity**	
Caucasian	22 (91.6)
Asian	1 (4.2)
Other	1 (4.2)
**Hispanic**	
No	24 (100)
**Participation in regular physical activity**	
Yes	12 (50)
No	12 (50)
**Income**	
<$23,000	1 (4.2)
$24,000–46,000	1 (4.2)
$47,000–70,000	6 (25)
>$71,000	15 (62.5)
Did not say	1 (4.2)
**Education**	
Some college	2 (8.3)
Associates/2-year degree	1 (4.2)
Bachelor’s degree	12 (50)
Graduate school+	9 (37.5)
**Marital status**	
Married	23 (95.8)
Widowed	1 (4.2)
**Weeks pregnant at loss**	
20–22	3 (12.5)
23–27	3 (12.5)
28–32	3 (12.5)
33–37	9 (37.5)
38+	5 (20.8)
Did not say	1 (4.2)

Women’s beliefs about physical activity after stillbirth were clustered around the following major categories: barriers to physical activity, benefits to physical activity, importance of physical activity, motivators for physical activity, and health care providers and physical activity. Sub-categories were created to allow for a more robust exploration of each category. Findings presented in results and discussion below are only those that are unique to women who have experienced a stillbirth and add to our knowledge in relation to the beliefs about physical activity specific to this population.

#### Barriers to physical activity

Barriers to physical activity participation included: emotional symptoms, lack of motivation, tired, guilt, pregnant body, and seeing other babies.

### Emotional symptoms and lack of motivation

A majority of women reported emotional symptoms and lack of motivation as barriers to physical activity participation after their loss, with emotional symptoms impacting their level of motivation. This was similar in women who met recommendations for physical activity as well as women who did not meet recommendations. Women reported that they were depressed and didn’t “feel like going for a walk” and that they were “emotionally drained”, which kept them from participating in physical activity. One woman said, “I don’t tend to be as active when I’m especially upset”. One woman who was active with yoga during her pregnancy reported that her emotional state kept her from practicing yoga after her stillbirth, “I tried doing yoga a few times but I had such a connection with yoga and with [child’s name]’s pregnancy … I emotionally could not do it”.

A woman who met physical activity recommendations at the time of the interview said, “…when I would be inactive it was because I physically just couldn’t get my mind where it needed to be to want to do anything”. Another woman who used the gym described the impact of seeing others at the gym, “Knowing that you might have to tell somebody, you might cry, and then you still have to do it. Sometimes it just makes you want to stay home”.

Interestingly, one woman described emotions associated with wanting to have another baby: “To be honest with you, I mean my reasoning is…what’s the point of getting back in shape when I want to be pregnant again right away… that’s just been hard for me to want to exercise just knowing that I want to get pregnant again”.

### Tired

Approximately half of the women mentioned being tired as a barrier to physical activity. Being tired was related to the lack of energy women had from feeling depressed, grief, and devastation.

One respondent characterized, “I was just so tired. I mean you know I’m sure it was a depression thing but I just, any sort of interaction with the real world just made me exhausted… just about the last thing I feel like I wanted to do was go back out and do something physical”.

### Guilt

One woman mentioned that guilt related to the loss of her baby was a major barrier to physical activity participation, “…When you are kind of dealing with all of this stuff it helps to do physical activity but at the same time when you get to a certain spot of just that despair and guilt you don’t really want to do anything…” In another instance, a woman who was not regularly active felt guilty about not being active and that her motivation for physical activity wasn’t like it used to be, “I sometimes feel a little guilty, I probably should be exercising more … motivation may not be there as it was before”.

### Pregnant body

A few women expressed barriers to participating in physical activity related to their pregnant bodies. A majority of these women were embarrassed about their pregnant bodies because they didn’t have a baby. “I’ve got this postpartum body but I don’t have a baby to show for it so, it was kind of, I was kind of embarrassed in the beginning because it looked like I just had a baby but I didn’t have a baby, you know,” one participant stated. Alternately, one woman didn’t want her body to change from its pregnant state, which kept her from being active: “I feel … there’s a little piece in there about not wanting my body to change …there’s something difficult about letting go of the body that was pregnant …”.

#### Seeing other babies

A few women reported that they didn’t want to see women with babies, which forced them to stay inside and limited being active (e.g., walking). One woman said, “A reason why I didn’t want to go out or exercise…everywhere you go there’s pregnant people, people with babies… after you lose a baby you don’t want to see that”.

#### Benefits to physical activity

Women reported that physical activity helped them to feel better mentally and emotionally, which helped them “cope” as they considered physical activity like “therapy”.

#### Feeling better mentally/emotionally

Many of the women reported that there were mental, emotional, and physical benefits of physical activity that helped them to feel better overall in relation to their loss. Women felt physical activity “boosts their mood”, gave them “more energy”, and helped them “feel better about yourself”. Women that were regularly active reported they used physical activity to manage their mental and emotional stress as a result of their loss. One respondent stated, “[Physical activity] just kind of puts my mind at ease a little bit. It kind of directs that negative stress that I have… puts them at the back of my mind”. Another woman shared, “…made me feel not so hopeless and worthless… It doesn’t get rid of the emotions completely but it makes me feel just a little bit better to get up the next day or finish out this day”. One woman felt activity helped her to be more mentally positive, “running… helps to clear my head, gives me the opportunity to think. I can think more positively rather than… with that negative tone”.

Even women who were not regularly active expressed that physical activity made a difference for them mentally. One woman said, “I know that I definitely feel better when I am active versus when I’m not. I have a better mindset, and I have better energy”.

#### Cope/therapeutic

Another benefit to physical activity was that was similar to therapy, and a means to cope. One woman stated, “I would say it’s probably my number one coping thing”. This was particularly true for women who met physical activity recommendations, as one woman pointed out, “I am dealing really well with my devastation and what happened and I tribute a lot of that to the fact that I am physically healthy and fit and I take care of my body”. A few women mentioned using it as treatment after their loss, “I know sort of in my head that…exercise is a great way to address feelings of sadness or anxiety it is sort of … treatment in a way”. Another said, “because of the grieving and being upset and losing [child’s name] it has even been more important to me because it clears my mind and it gives me that opportunity to think”. One woman also reported that physical activity played a major role in her life after the loss: “Exercise… was a big coping factor after”.

#### Importance of physical activity

Most women reported that physical activity was important to them (regardless of if they were currently active or not). The few women who believed that physical activity wasn’t important expressed that it never had been important (i.e., prior to the stillbirth) or because after the loss it became less important (i.e., not a priority anymore). Reasons for physical activity being important included working through grief and spending time with themselves.

### Grief

Women reported that their grief was “horrible” but that physical activity was important for working through grief. Physical activity was an outlet for them and allowed them time to process grief. This was mostly true for women who met guidelines for physical activity. Women characterized the role of physical activity in dealing with grief in several ways, including, “It helped with the grief of the loss really so it was an outlet for that,” “It’s a stress relief and a way to manage my grief,” and “It allows me some physical expression of grief, a physical outlet”.

#### Time for self

Another importance of physical activity for women after loss was time for self. Women viewed physical activity as a time to be alone, reflect, and focus on self. One woman felt physical activity was more important than prior to her loss, “an hour of my time to focus on myself and now it’s really, it’s even more important for me”. Another woman walked a lot right after her loss, “…walked a little more because it wasn’t about the physical activity it was about kind of trying to get away… longer walks, probably slower walks, not quite as focused on the physical activity part but … the getting away part”.

#### Motivators for physical activity

Women were motivated for physical activity because they wanted to have more children, were concerned about their body shape, wanted to be a role model to other women, and because they were active prior to having a stillbirth.

### More children

Regardless of current activity levels, almost half of the women reported being motivated for physical activity because of the desire to have another child. Women expressed that they needed to be healthy to start “trying again”. “I’m trying to get down to the weight I was before and as healthy as I was before so we can try again as soon as we can”. Women were motivated to be in their best shape for a pregnancy and knew that physical activity participation would help them do this. One woman said, “Physically I want to be in the best [shape], I want to give it the best chance I can so I have to be physically ready to carry another baby”. Another woman said, “I think the thing that makes me feel the best about exercising is thinking about being strong and being healthy and wanting to have another child and being successful about that …”.

#### Body shape/weight

A majority of women were motivated to participate in physical activity because of their dissatisfaction with their current body shape/weight, which often stemmed from women wanting to return to their pre-pregnancy body shape/weight and/or not feeling comfortable in their current body following the pregnancy. “I just wanted to get rid of the weight because I no longer, I had nothing to show, … I thought I need to get rid of this weight because I don’t have a child, there’s no excuse for me to stay the way I am”. Another woman said, “I guess the personal motivation is I haven’t lost a lot of the baby weight … I’m not comfortable with my appearance…”. Women who were meeting physical activity recommendations seemed to use the desire to get their “weigh off” as the catalyst to being active. Additionally these women were concerned with weight maintenance once they were able to return to their pre-pregnancy body shape/weight. Weight maintenance was a motivator for some, in addition to being “back in shape” and having things “fit better”.

### Role model

There were a few women who mentioned that being a role model to their existing children and/or other newly bereaved women motivated them to be active. Women said that they “wanted to be a good role model for the children that they did have”. They also said that they wanted others to see them, “dealing with grief and not succumbing to it” and this helped them to be motivated for activity.

#### Already an exerciser

A few women mentioned that their motivation for physical activity after their loss stemmed from being an avid exerciser prior to their loss. These women shared that being active was a part of who they are and that they couldn’t imagine going through [stillbirth] without physical activity. It was the “previous experience of knowing that exercise feels good” that helped them to be active.

#### Health care providers and physical activity

No women reported that their health care providers prescribed physical activity after their loss. Most women reported that their health care providers only referred to activity in the context of recovering and that they were encouraged to “return to normal activity” sometime after their delivery (time varied between participants). One woman said, “That was it, there was nothing. I went in… to make sure I was healing ok from the stitches and everything… my OB had said you can do whatever you were doing before”. Another woman shared, “I think they told me the standard, ‘don’t do anything too strenuous until your six week checkup’”. Women who were regularly active and specifically asked about returning to activity were also not given any advice beyond, ‘whenever your body feels ready’. One woman said, “He said whenever I felt ready to start working out I could go back at it. He said my body would tell me if it was not ready”.

Women mentioned that it would have been helpful to have guidance from their health care provider and that perhaps such advice would have motivated them to be active, “I think it would have been easier if my doctor gave me information maybe that would’ve helped me motivate a little bit”. Another woman shared, “Wish my health care provider would have provided more information about physical activity”. A few women reported that they were provided written materials that included some information about physical activity. One woman was given an article relative to loss: “…and there were probably 10 things on there … number one, two, and three were get enough sleep, eat healthy, and exercise”. Another woman said she was given “…the same pamphlet they give to moms who have a living baby, just that you take it easy for a little while, but you should be able to resume physical activity when you feel able”.

## Discussion

This is the first study to qualitatively explore the beliefs about physical activity in women after a stillbirth. Not surprisingly, women reported a lack of motivation due to the emotional symptoms they were experiencing from their loss as a barrier to physical activity participation. Even women who considered themselves regularly active (~50%) reported a lack of motivation for physical activity due to emotions, especially immediately following the stillbirth. Physical activity may be a useful tool to help women who have experienced stillbirth work through their emotional symptoms and has been recognized as a means to improve depressive symptoms in women with post-partum depression and at ‘high risk’ during pregnancy [18-22]. However, women who have experienced a stillbirth may need to be educated about the positive effects that physical activity could have on their mental and emotional health. Understanding that physical activity may be an outlet for the emotions that they are feeling may motivate them to participate in physical activity [23,24]. Additionally, support systems at hospitals may need to provide women resources for physical activity (i.e., yoga, group exercise classes) in addition to treatment/support groups that are typically referred to women who have experienced loss. Future research is warranted to assess the differences in physical activity perceptions of women who have experienced stillbirth that were active prior to and during their pregnancy as compared to women that were inactive.

Women reported that feeling guilty kept them from participating in physical activity. Other studies in healthy women have reported that guilt is a barrier to physical activity because it takes time away from family and/or work [25,26]. Some women in this study felt guilty for the same reasons; however, some reported feeling guilty about their actual loss (e.g., could I have done something differently, what do others think of me now) and this guilt, or “despair”, from the loss kept them from being active. It is important that health care providers consider that women may feel guilty about having a stillbirth and that women are provided resources for counseling and made aware of the importance of taking care of themselves (i.e., general health, quality of life) and the role that physical activity may play in taking care of their overall health.

Women also reported their pregnant bodies were barriers to physical activity. Women didn’t want people to see their pregnant bodies and answer questions about what happened (e.g., Where is the baby?). Conversely, some women didn’t want to lose their pregnant body because it was all they had to represent the baby they had lost. Recent studies suggest that pregnancy associated weight gain and failure to lose weight after giving birth may potentially increase risk for chronic disease later in life (e.g., overweight, obesity) [27,28]. Additionally, as evident in women with post-partum depression, depressive symptoms in women who have experienced loss may inhibit them from participating in physical activity necessary to lose weight [27-29]. Special support for physical activity, such as private places to be active (e.g., cardiac rehab “type” classes in a hospital or community center) and physical activity specialists who have experience working with bereaved patients, may be necessary for women after loss. Additionally, home-based exercise programs may be a viable option for women after loss. There is a need for further research in this area.

Women also didn’t want to see other women with babies and this kept them from being active. The emotions associated with seeing other babies was difficult and women preferred to stay inside rather than be active (e.g., walk), despite knowing the benefits of physical activity. These findings further justify the need for more ‘private’ avenues for women to be active, especially immediately after their loss.

Women felt that physical activity was beneficial for their mental and emotional health and was a means of coping or therapy. Physical activity may provide an avenue for women to manage depressive symptoms and take care of themselves outside of a hospital or health care provider setting. Currently, there is no standard of care for women after stillbirth [29]. Mostly women are prescribed psychiatric mediations for acute grief and/or provided loss support group resources in addition to their scheduled 6-week check-up. None of these ‘treatments’ represents a way to teach women how to adaptively cope with grief and depressive symptoms they may experience from their loss. Women in this study, mostly those that were already active, reported using physical activity as a treatment and to psychologically feel better. These findings provide rationale that physical activity programs offered through hospitals or other community-based settings may represent a potential alternative strategy to help women cope with loss. More research in this area is warranted.

Similar to the benefits of physical activity reported by women in our study, women reported that physical activity was important to them to work through grief and to spend quiet time for themselves. This provides further justification of the need for research using physical activity as an alternative strategy to help women cope with their loss. Particularly, due to its ability to help individuals self-reflect and quiet their mind, yoga may be an attractive form of physical activity for women who have experienced stillbirth. Yoga is the practice of integrating physical postures and movement with controlled breathing and meditation [18-22] and is known to improve depressive symptoms and quality of life [20-22]. In pregnant women, yoga has been established as a feasible and efficacious way to relieve stress and anxiety [30]. Yoga represents an innovative, mindful strategy that could easily be disseminated within hospital or community based programs to improve depressive symptoms in women who have experienced stillbirth. There is a need to conduct physical activity interventions in women after loss, specifically utilizing yoga.

Much of the motivation for physical activity participation was because women wanted to have more children and were concerned about being healthy for another pregnancy. It has been clearly established that a healthy weight during pregnancy improves health outcomes for both the mother (e.g., less risk for preeclampsia, gestational diabetes, and high blood pressure) and the baby (e.g., healthy birth weight, less risk for pre-term birth) [29,31,32]. Women in our study understood that physical activity participation was necessary to achieve a healthy weight, despite their current activity levels. Pregnancy has been considered a time in a woman’s life in which she may be motivated to change health behaviors and thus represents an important time to promote adoption of physical activity behaviors [33-35]. This may be especially true for women who have experienced loss as they not only have a desire to have more children but may be more concerned for the healthy development of the fetus as compared to women who have had live, healthy births [15,30,34,36]. It is recommended that women wait six months after a stillbirth to get pregnant again [5,37]. Health care providers could use this time to assist women in establishing healthy behaviors, namely regular physical activity participation, to lose weight and be prepared for their subsequent pregnancy. Additionally, behavior strategies could be utilized (i.e., goal setting, self-monitoring) to help improve the likeliness that a woman is able to maintain physical activity after their subsequent pregnancies.

A few women were motivated for physical activity participation because they wanted to be a role model to support other women and ‘show’ that they don’t have to ‘succumb’ to grief. Research suggests that support for women after loss may be most credible when it comes from others who have experienced loss [38]. This has very important implications for physical activity programming in women who have experienced loss. Social support is a well-known determinant for physical activity participation in women [39]. Using a role model/social support approach to promote physical activity could be a viable way to help women who have experienced loss adopt and maintain physical activity participation [40]. Hospitals could pair women based on time since loss, helping women who may have more recently experienced loss participate in activity with a woman who is farther from time since loss. This is further justified with women in our study reporting being motivated for physical activity because they were active prior to loss and had experienced the emotional benefits to physical activity. These same women could participate in the role model/social support approach. More research is warranted using a role model/social support approach.

One of the most important findings from this study was the information gathered related to health care providers and physical activity participation. Health care providers are not providing information (e.g., exercise prescription, programming) relative to physical activity for women who have experienced loss. This is not surprising, as studies report that women may not receive advice or adequate information about physical activity during their health care provider visits because health care providers are often constrained by time and may lack the knowledge about proper physical activity recommendations [33,41-44]. Research suggests health care providers are uncomfortable with death and bereavement, particularly related to infants and children [45]. Taken together, women who have experienced loss are not receiving any information from their health care providers about the importance of physical activity participation for their physical (e.g., weight) mental, and emotional health. There is a desperate need to better educate and train health care providers to provide recommendations and guidance for physical activity participation. This is especially true in women who have experienced loss due to the positive health implications (i.e., improvement in depressive symptoms, achieve healthy weight for subsequent pregnancies) of physical activity participation.

### Limitations

Although this study fills an important gap in the literature, contributing much needed information about the potential of physical activity as a “treatment” for women who have experienced stillbirth, there are a few limitations to be noted. First, participants for this study were predominantly educated, married, Caucasian women. Findings for this study may not be generalizable to a diverse population of women. Second, women commented on their physical activity participation in the interviews (self-reported) and this has inherent limitations (e.g., recall, misrepresented information). Future research should include objective activity monitors (accelerometer, body media) to measure physical activity in this population.

## Conclusion

This study was the first to qualitatively explore the beliefs about physical activity in women after a stillbirth. Findings from this study may help to improve the health of women who have experienced stillbirth. There is no standard of care for women after loss. Beyond the ‘treatments’ currently offered (e.g., psychiatric medicine, support groups), physical activity may offer women a means for: 1) self-managing grief and emotions associated with loss on a daily basis, 2) improving health for subsequent pregnancies, and 3) improving quality of life long-term. Future research in this area is highly warranted.

## Competing interests

None of the authors have a any financial or non-financial competing interests related to the publication of this manuscript.

## Authors’ contributions

JLH has made substantial contributions to the conception and design, training and managing for data collection, entry and analysis, drafted the manuscript and revised according to other author’s comments and contributions. JC participated in data entry, analysis, and drafting of the manuscript. KR conducted data collection, participated in data entry and analysis and drafting of the manuscript. SW participated in conception and design of the study, recruitment of participants for data collection, and drafting the manuscript. All authors participated sufficiently in the work to take public responsibility for appropriate portions of the content. All authors read and approve the final manuscript.

## Pre-publication history

The pre-publication history for this paper can be accessed here:

http://www.biomedcentral.com/1471-2393/14/26/prepub
